# Exploring the Use of Hospital and Community Mental Health Services Among Newly Resettled Refugees

**DOI:** 10.1001/jamanetworkopen.2022.12449

**Published:** 2022-06-02

**Authors:** Soumya Mazumdar, Shanley Chong, Sandy Eagar, Stephanie Fletcher-Lartey, Bin Jalaludin, Mitchell Smith

**Affiliations:** 1 Population Health Intelligence, South Western Sydney Local Health District, Liverpool, Australia; 2 South Western Sydney Clinical School, University of New South Wales Medicine, Liverpool, Australia; 3 New South Wales Refugee Health Service, Liverpool, Australia; 4 Burnet Institute, Melbourne, Australia; 5 School of Population Health, University of New South Wales Medicine, Kensington, Australia

## Abstract

**Importance:**

Resettled refugees in high-income countries represent a vulnerable population. It is known that refugees have high rates of trauma-related mental health issues; however, ad hoc research has generally revealed low rates of health services use among refugees. Such research usually samples a population at a single point in time and is based on targeted surveys. Because refugee populations change over time, such research becomes expensive and time-consuming for agencies interested in routinely publishing statistics of mental health services use among refugees. The linking of large administrative data sets to establish rates of use of mental health services among resettled refugees is a flexible and relatively inexpensive approach.

**Objective:**

To use data linkage to establish rates of mental health services use among resettled refugees relative to the general population.

**Design, Setting, and Participants:**

This cross-sectional study implemented data linkage from the Refugee Health Nurse Program for 10 050 refugees who resettled in Sydney, Australia, from October 23, 2012, to June 8, 2017, with data concerning use of community mental health services and mental health hospitalization from New South Wales Health databases. Data were analyzed between June 1, 2019, and December 31, 2021.

**Main Outcomes and Measures:**

Rates of service contacts with community mental health services among the resettled refugees were compared with those of the general population by age, sex, and the most common *International Statistical Classification of Diseases and Related Health Problems, Tenth Revision*, diagnosis codes. Length of community mental health service sessions and rates of mental health hospitalizations were also compared.

**Results:**

Among the 255 resettled refugees who had contacts with community mental health care services and were not missing data (median age, 35 [range, 4-80] years; 117 [64%] male and 138 [54%] female), 153 (60%) were born in Iraq and 156 (61%) were Arabic speaking. This population was less likely to use mental health services than the general population and had shorter community mental health consultations. The rate of contacts with community mental health services for depressive disorders among the resettled refugee population was 40% (95% CI, 33%-46%) lower than that among the general population. Rates of same-day hospitalization per 10 000 person-years were not significantly different between the refugee population (4 [95% CI, 2-8]) and the general Australian population (7 [95% CI, 7-7]). However, the refugee population was 17% (95% CI, 6%-29%) more likely than the general Australian population to interact with the community mental health system for severe stress- and adjustment disorder–related diagnoses.

**Conclusions and Relevance:**

These findings suggest that refugees who have resettled in Australia tend to use fewer mental health services than the general population except for services devoted to stress- and adjustment disorder–related diagnoses. These findings also suggest that it is possible to successfully leverage data linkage to study patterns of mental health services use among resettled refugees.

## Key Points

**Question:**

How do rates of use of community and tertiary mental health services among a representative refugee cohort compare with benchmark rates in a high-income country?

**Findings:**

This cross-sectional data linkage study of 10 050 refugees in Australia found that resettled refugees used fewer community mental health and hospitalization services overall than the general population. However, service contacts with community mental health services with a primary diagnosis of reaction to severe stress and adjustment disorders were 17% higher than in the general population.

**Meaning:**

These findings suggest that resettled refugees use fewer mental health services than the general population, except for specific diagnoses.

## Teaser

This cross-sectional data linkage study compares rates of mental health services use among resettled refugees in the Refugee Health Nurse Program of South Western Sydney with those of the general population in Australia.

## Introduction

Resettled refugees in high-income countries often experience mental health issues, which may translate into higher rates of mental health services use.^1^ Refugee countries of origin change over time,^2^ rendering refugee health studies—many of which are ad hoc studies based on targeted surveys—less relevant. In addition, study populations may be small, attribute refugee status indirectly, or lack an appropriate comparison or benchmark population.^3^

Australia has traditionally received refugees from multiple regions of the world, and studies of refugee health have had many of the above limitations. Refugee mental health studies are usually based on survey data,^4^ with 1 study^3^ indirectly attributing refugee status to routinely collected data. However, refugee populations entering Australia have seen changes in composition over time. Thus, previous studies of refugees from Vietnam, Somalia, or former Yugoslavian republics^3,4^ may not apply to the current refugee population. Although an in-depth discussion on the current refugee population is beyond the scope of this study, the incoming refugee population in Australia during the period 2012 to 2017 was predominantly Syrian or Iraqi. These refugees faced displacement from war in their countries of origin and may have lived in camps—furthering their trauma—and with relatively large families,^5,6^ hence the need for a cost-effective and timely means of collecting, collating, and measuring the rate of mental health services use by refugees and comparing it with use by the general Australian population.

Many mental health services such as community mental health services or public hospital stays are free to Australian citizens and humanitarian visa holders (ie, refugees). In Australia, from 2018 to 2019, the mental health service landscape encompassed 10 million community mental health service contacts, 275 000 overnight and 59 888 same-day psychiatric hospitalizations, 106 401 psychiatry disability service contacts, 271 040 psychiatric emergency presentations, and 8000 residential care contacts.^7^ Cases with severe emergency mental health presentations are likely to be hospitalized, and very few refugees are likely to be in disability services or residential care,^8^ making community mental health services and hospitalizations the most salient for an investigation of refugee mental health services use.

In addition, refugees in New South Wales (NSW) have access to free specialized trauma services (Service for the Treatment and Rehabilitation of Torture and Trauma Survivors [STARTTS]).^8^ STARTTS is a statewide specialized service, and according to correspondence from STARTTS, most clients start using their services 3 years after their arrival in Australia (T. Carlena, BSc, email communication, April 2021).

Linking data sets of resettled refugee populations with routinely collected health data may be a viable approach to measuring refugee mental health use. Routinely collected data are used to publish mental health service use statistics for the general Australian population. Data on refugees can be linked to the databases that inform these statistics to generate valid comparisons. This research linked a large representative data set of NSW Refugee Health Service recipients to NSW community mental health use and mental health hospitalization data. Our research question was: How do the rates of community and tertiary mental health services use among a representative refugee cohort compare with Australian benchmark rates?

It is also important to note that in the context of this study, the term *refugee* denotes a resettled refugee or a refugee who has been granted permanent residency status through the humanitarian visa program of the Australian government (visa details available in eAppendix 1 in the Supplement). This excludes refugees who have been refused permanent residency in Australia and those seeking legal review, refugees in detention, or other refugees holding temporary visas. From 2019 to 2020, 13 171 humanitarian permanent residency visas were granted^9^ and approximately 1300 refugees were in detention.^10^ As of December 2019, approximately 83 000 individuals were either awaiting the results of their asylum application or had been refused residency and were awaiting deportation. Past research has shown that refugees with temporary visas and in detention experience anxiety, depression, and posttraumatic stress disorder (PTSD).^11,12^ Although studying all refugee groups is essential, this study focuses explicitly on refugees with permanent visas and their engagement with the Australian mental health system. Hereinafter, we use the term *individuals* to refer to resettled refugees wherever feasible to avoid unnecessary labeling. We refer to the comparison population as the Australian population or general Australian population.

## Methods

This cross-sectional data linkage study was approved by the NSW Population and Health Services Research Ethics Committee. Waiver of informed consent was granted because of a number of reasons, including the infeasibility of obtaining consent and strong data deidentification protocols followed by the researchers. We followed the Strengthening the Reporting of Observational Studies in Epidemiology (STROBE) reporting guideline.

Our base data are from Refugee Health Nurse Program (RHNP)^13^ health clinics in South Western Sydney, which screened approximately 40% of all newly arrived refugees (<1 year) from October 23, 2012, to June 8, 2017 (n = 10 050) who resided in the greater Sydney metropolitan area. Sydney accounts for 20% of Australia’s population and received approximately 28% of all humanitarian settlers in Australia from 2009 to 2014.^14^ The data included basic demographics and were validated against settlement data from the Department of Settlement Services and compared with the general Australian population (eAppendix 1 in the Supplement). Although the Department of Settlement Services data (eAppendix 1 in the Supplement) also provide a demographic overview of the total Sydney resettled refugee population, we could not ascertain the demographics of the refugees not screened by RHNP because data linkage between Department of Settlement Services and RHNP was not a feasible option.

We linked RHNP records to 2 separate NSW Health data sets: the Mental Health Ambulatory Care Service data collection and the NSW Admitted Patients Data Collection for the 2012-2017 period (details are provided in eAppendix 2 in the Supplement). The Mental Health Ambulatory Care Service data collection covers all contacts with publicly funded nonadmitted community mental health care services (CMHCS), including hospital outpatient and ambulatory mental health clinic visits and home visits. The NSW Admitted Patients Data Collection covers all hospitalizations in private and public hospitals. We use the term *CMHCS* hereinafter to refer to the Mental Health Ambulatory Care Service linked data.

The Australian Institute of Health and Welfare (AIHW) routinely publishes CMHCS service contact and mental health hospitalization statistics collated from various states for the Australian population, derived from the same databases as above (CMHCS and NSW Admitted Patients Data Collection). These data serve as an appropriate benchmark for comparing statistics of use of RHNP mental health services. To retain comparability, we followed AIHW standards, categorizations, and definitions. Thus, we used AIHW’s reporting unit of CMHCS data, which is the service contact. A service contact is defined as a clinically significant service by a specialized mental health service professional such as a nurse or a psychologist.^7^ Each service contact is associated with a primary diagnosis (coded in *International Statistical Classification of Diseases and Related Health Problems, Tenth Revision* [*ICD-10*]), and 1 individual can have multiple contacts and/or diagnoses.

We used only public hospital data following AIHW standards. Although 60% of all hospitalizations in Australia are in public hospitals, public hospitals are free to permanent residents, citizens, and humanitarian visa holders. Therefore, refugees use the free public hospital system rather than the private hospital system, which requires out-of-pocket payments or health insurance.^15^ The AIHW statistics on hospitalization are reported as same-day and overnight stays, and we followed this convention. Mental health hospitalizations were identified using a reference list of mental health primary diagnosis *ICD-10* codes published by AIHW^7^ (eAppendix 2 in the Supplement).

Our first goal was to compare RNHP cohort service contacts with CMHCS relative to the general population’s service contacts with CMHCS by age group, sex, and primary *ICD-10* diagnosis. To achieve this goal, we used standardized ratios, which were calculated as the observed number of RHNP cohort service contacts in a specific age, sex, and/or diagnosis category divided by the expected number of service contacts. The method used to calculate the standardized ratios is provided in eAppendix 2 in the Supplement.

The RNHP cohort sessions with the CMHCS had start and end times encoded, allowing us to evaluate service contact session lengths by client present or absent status (indicating whether the patient was physically present or communicated via phone) and compare these with AIHW session length statistics for the general Australian population. Finally, crude rates of mental health hospitalizations in the RHNP cohort were compared with AIHW statistics. We also compared rates of same-day and overnight hospitalization both with and without specialized care, in addition to the length of stay for overnight hospitalizations. Any statistic based on fewer than 10 individuals has been marked out in our results and should be interpreted with caution. We have provided 95% CIs where possible. For session lengths and hospitalizations, comparisons were made between the RHNP cohort and the Australian population when samples were of sufficient size (n ≥ 10) using the 2-sample test of proportions and *z* scores (2-sided test) with significance set at *P* < .05. All analyses were implemented on data obtained between October 23, 2012, and June 8, 2017, using R, version 3.6.0 (R Project for Statistical Computing).^16^ Data were analyzed between June 1, 2019, and December 31, 2021.

## Results

Of the 10 050 individuals in the RHNP cohort, 302 (3.0%, including those with missing data) used CMHCS services, and 78 (0.8%) were hospitalized with a psychiatric diagnosis. For those using CMHCS services and not missing data (n = 255), ages ranged from 4 to 80 years, with a median of 35 years (117 [64%] male and 138 [54%] female). Arabic was spoken by 156 individuals (61%) and Farsi was spoken by 43 individuals (17%); overall, 22 languages were spoken. There were 19 different countries of birth, with Iraq being the most common (153 [60%]). There was a mean of 22 CMHCS service contacts per person, compared with 25 service contacts in the NSW population, and 21 service contacts per person in the Australian population in 2016.^7^ The CMHCS clients in the RHNP cohort made a median of 1 service contact a week. Although just 1 individual used CMHCS during the entire study period (approximately 5 years), 23 (9%) had service contacts with the CMHCS just once during this period. The median time between first and last visit was 80 days. All RHNP cohort age groups used CMHCS less than the Australian population, with only the group aged 25 to 34 years using services at a rate comparable to that of their Australian counterparts (standardized ratio, 0.92 [95% CI, 0.87-0.97]). Although both men and women in the RHNP cohort had lower service contact rates than the Australian population, men in the RHNP cohort had lower rates than women (standardized ratios, 0.49 [95% CI, 0.47-0.51] vs 0.66 [95% CI, 0.64-0.69]) (Table 1).

**Table 1.** Service Contacts Among the RHNP Cohort to Community Mental Health Care Services by Age, Sex, and Principal Diagnoses Compared With the Australian Population

Characteristic	Australian population		RHNP cohort		Crude rate per 10 000 person-years in Australian population	No. of expected service contacts in RHNP cohort^a^	Standardized ratio (95% CI)
	No. of service contacts	Person-years	No. of service contacts	Person-years			
Age group, y							
0-17	4 476 820	21 534 609	729	8476	0.21	1762	0.41 (0.39-0.45)^b^
18-24	8 883 882	8 477 837	304	2902	1.05	3041	0.10 (0.09-0.11)^b^
25-34	4 920 842	14 059 558	1395	4339	0.35	1519	0.92 (0.87-0.97)^b^
35-44	8 342 382	13 045 220	845	3544	0.64	2266	0.37 (0.35-0.40)^b^
45-54	8 615 744	12 524 497	1054	2609	0.69	1795	0.59 (0.55-0.62)^b^
55-64	6 270 451	10 882 192	265	1696	0.58	977	0.27 (0.24-0.31)^b^
≥65	2 820 501	14 042 295	220	1462	0.20	294	0.75 (0.65-0.85)^b^
Grand total	44 330 622	94 566 208	4812	25 028	0.47	11 733	0.41 (0.40-0.42)^b^
Sex							
Male	17 367 628	47 365 335	2279	12 744	0.49	4673	0.49 (0.47-0.51)^b^
Female	15 004 485	47 200 873	2535	11 994	0.66	3813	0.66 (0.64-0.69)^b^
*ICD-10* diagnosis code (diagnosis)							
F29 (unspecified nonorganic psychosis)	93 616	24 308 689	166	25 028	0.004	96	1.72 (1.48-2.01)^c^
F23 (acute and transient psychotic disorders)	67 989	24 308 689	108	25 028	0.003	70	1.54 (1.27-1.85)^c^
F43 (reaction to severe stress and adjustment disorders)	322 517	24 308 689	388	25 028	0.01	332	1.17 (1.06-1.29)^b^
F32 (depressive episode)	550 042	24 308 689	340	25 028	0.02	566	0.60 (0.54-0.67)^b^
F41 (other anxiety disorders)	259 593	24 308 689	132	25 028	0.01	267	0.49 (0.42-0.58)^c^
F31 (bipolar affective disorders)	341 381	24 308 689	101	25 028	0.01	351	0.29 (0.24-0.35)^c^
F20 (schizophrenia)	1 474 023	24 308 689	126	25 028	0.06	1518	0.08 (0.07-0.10)^c^
F99 (mental disorder, not otherwise specified)	2 674 553	24 308 689	108	25 028	0.11	2754	0.04 (0.03-0.05)^c^
99 (mental diagnosis yet to be allocated)^d^	NA	NA	3159	25 028	NA	NA	NA

Abbreviations: *ICD-10*, *International Statistical Classification of Diseases and Related Health Problems, Tenth Revision*; NA, not applicable; RHNP, Refugee Health Nurse Program.

^a^ Indicates the number of service contacts that would be expected in the RHNP cohort if they were having service contacts at the same rate as the Australian population.

^b^ Significant at *P* < .05.

^c^ Based on service contacts made by fewer than 10 individuals.

^d^ Not an *ICD-10* code; used by community mental health care services when a mental health diagnosis has not been allocated to a given service contact.

Thirty-two individuals had multiple diagnoses, with 29 individuals having 2 diagnoses and 3 individuals having 3 diagnoses. Of those with multiple diagnoses, almost one-third had reaction to severe stress (*ICD-10* code F43) or PTSD as one of the comorbid conditions. However, 46 unique separate diagnoses were present within individuals with comorbid diagnoses. When standardized ratios were compared across *ICD-10* diagnosis codes, we observed that the RHNP cohort was less likely to use services for most diagnoses than the Australian population, with service use for schizophrenia being 92% lower (standardized ratio, 0.08 [95% CI, 0.07-0.10]). However, many of the diagnostic group statistics were based on service contacts made by fewer than 10 individuals and may therefore be unreliable. The 2 diagnoses based on a sufficient number of individuals with rates significantly different from those of the Australian population were depressive episodes (n = 20) and reaction to severe stress and adjustment disorders (n = 43). The RHNP cohort was 17% (95% CI, 6%-29%) more likely than the Australian population to have a service contact with a primary diagnosis of reaction to severe stress and adjustment disorders (standardized ratio, 1.17 [95% CI, 1.06-1.29]) but 40% (95% CI, 33%-46%) less likely than the Australian population to have a service contact with a primary diagnosis of a depressive episode (standardized ratio, 0.60 [95% CI, 0.54-0.67]) (Table 1).

When session times were analyzed, the RHNP cohort was significantly more likely to have shorter sessions (≤30 minutes) than the Australian population (Table 2). This was generally true irrespective of whether the session was face-to-face or otherwise.

**Table 2.** Session Duration by Client Presence or Absence

Duration	Client present, % (95% CI)^a^		Client absent, % (95% CI)	
	Australian population	RHNP cohort	Australian population	RHNP cohort
<5 min^b^	0.6 (0.6-0.7)	0.4 (0.3-0.7)	9.3 (9.2-9.3)	7.5 (6.8-8.2)
5 to 15 min	32.2 (32.2-32.3)	40.7 (39.2-42.1)^c^	50.7 (50.7-50.8)	59.8 (58.4-61.0)^c^
16 to 30 min	24.0 (24.0-24.0)	30.4 (29.1-31.8)^c^	22.7 (22.6-22.7)	23.0 (21.9-24.2)
>0.5 to 1 h	25.3 (25.3-25.3)	20.8 (19.7-22.1)^c^	11.7 (11.7-11.7)	7.1 (6.5-7.9)^c^
>1 to 3 h	16.2 (16.1-16.2)	7.2 (6.4-8.0)^c^	5.1 (5.1-5.2)	2.5 (2.1-3.0)^c^
>3 h^b^	1.7 (1.7-1.7)	0.5 (0.7-0.7)	0.5 (0.5-0.5)	0.1 (0.02-0.2)
All	100	100	100	100

Abbreviation: RHNP, Refugee Health Nurse Program.

^a^ Implies the individual was physically present or otherwise in communication during the service contact.

^b^ Estimates are based on multiple service contacts from fewer than 10 individuals.

^c^ Significant differences between RHNP cohort and Australian population (2-sample test of proportions *z* score, 2-sided test) at *P* < .05.

The RHNP cohort had 115 mental health hospitalizations representing 47 individuals. Of these 115 hospitalizations, 48 (42%) were same-day admissions and 67 (58%) were overnight hospitalizations (Table 3). The numbers were small, and the overall rates of same-day hospitalizations among the RHNP cohort (19 [95% CI, 14-25] per 10 000 person-years) were not significantly higher than those among the Australian general population (24 [95% CI, 24-24] per 10 000 person-years), with rates of overnight hospitalizations being significantly lower (27 [95% CI, 21-34] vs 98 [95% CI, 98-98] per 10 000 person-years). However, rates of overnight hospitalizations requiring specialized care remained significantly higher among the RHNP cohort (22 [95% CI, 17-29] vs 10 [95% CI, 10-10] per 10 000 person-years). The mean length of stay among the RHNP cohort was not significantly different from that of the Australian population (20 [95% CI, 13-28] vs 16 days). Also, approximately one-third of the RHNP cohort using CMHCS were hospitalized with a mental health–related primary diagnosis (rate, 22 [95% CI, 17-29] per 10 000 person-years). Conversely, approximately two-thirds of those hospitalized with mental health diagnoses had a service contact with the CMHCS a median of 17 days before being hospitalized.

**Table 3.** Mental Health Hospitalizations Among the RHNP Cohort by Age and Sex Compared With the Australian Population^a^

Hospitalization type	No. in RHNP cohort	Hospitalizations per 10 000 person-years (95% CI)	
		Australian population	RHNP cohort
Same day			
Overall	48	24 (24-24)	19 (14-25)
Not requiring specialized care	38	16 (16-16)	15 (11-21)
Requiring specialized care	10	7 (7-7)^b^	4 (2-8)
Overnight			
Overall	67	98 (98-98)^b^	27 (21-34)
Not requiring specialized care	12	22 (22-22)^b^	5 (3-9)
Requiring specialized care	55	10 (10-10)^b^	22 (17-29)

Abbreviation: RHNP, Refugee Health Nurse Program.

^a^ Includes hospitalizations in public hospitals only.

^b^ Significant differences between RHNP cohort and Australian population (2-sample test of proportions *z* score, 2-sided test) at *P* < .05.

## Discussion

We present one of the most extensive studies of use of mental health services by refugees, to our knowledge, using linked data. We found that rates of mental health services use were significantly lower and service contact sessions were shorter among resettled refugees than among the general Australian population. Rates of service use for diagnoses related to severe stress and adjustment disorders (which encompass PTSD) were significantly higher among refugees than among the Australian population; however, some individuals may have missed a diagnosis when using CMHCS.

This study has several strengths. First, it is one of the largest studies of refugee mental health, representing approximately 40% of refugees who settled in Sydney from 2012 to 2016. For comparison, the largest reported study of refugee mental health to date included 1161 participants.^1,4^ Second, we used metrics directly comparable with published national rates, enabling comparisons with a nonrefugee population. Third, the CMHCS and NSW Admitted Patients Data Collection data sets are comprehensive in that they capture all community mental health visits and public hospitalizations in NSW.

We found significantly lower rates of use of CMHCS among the RHNP cohort than among the Australian population, confirming a previously described pattern of health services use among refugees in Australia and elsewhere.^3,4,17,18^ Lower use of mental health services is reflected in the number of service contacts and mental health hospitalizations and the time spent during mental health sessions, with refugees being overrepresented in shorter sessions than the Australian population.

There are several reasons why refugees may have lower rates of mental health services use, some of which may reflect lower rates of prevalence of conditions such as schizophrenia. This is because refugees with severely limiting conditions such as schizophrenia are unlikely to make the long, arduous, and complicated journey from their country of origin to the host country. However, for other conditions, lower use may not reflect lower prevalence. Instead, lower use may be driven by stigma, ignorance, misidentification or somatization of psychiatric symptoms, ignorance of existing support services and referral chains, use of religious sources of mental health support (including holy books and religious leaders), unwillingness to revisit traumatic memories, and avoidance of emotional discomfort.^19,20^ Other reasons could be a lack of time and/or financial resources, language and/or cultural barriers, unstable housing, or a lack of trust.^21^ A study in Australia^22^ reported that two-thirds to one-half of all refugees do not seek help for emotional problems. Although CMHS visits were significantly lower, the association with hospitalization was less clear, with overall no difference in mental health hospitalization between the RHNP cohort and the Australian population. This could reflect unmet need, because hospitalizations for mental health represent relatively severe mental health needs that primary care services have not met. Service contact sessions with refugees are generally shorter than those with the Australian population, which could be an instance of what some researchers call the “inverse care law,” in which care provisioning is the inverse of the required need. Researchers from the UK and Australia have found an inverse association between socioeconomic deprivation and length of primary care sessions.^23,24^ Different mechanisms may be responsible for this association and, in the context of refugee health services, remain an area of future enquiry. It is also important to note that one-third of individuals with mental health hospitalizations interacted with the CMHCS, indicating a reasonable degree of access to primary care before hospitalization.

Our study found significantly high rates of health services use due to reaction to severe stress in the RHNP cohort. High levels of trauma-related illnesses among refugees are well documented in the literature.^1,25^ Prevalence rates of PTSD among refugees, in general, have been reported to be 9% (99% CI, 8%-10%).^1^ A Western Sydney study among Iraqi refugees reported a prevalence of 31.1% for PTSD and 39.1% for severe psychological distress.^26^ In the RHNP cohort, less than 1% used CMHS services with a primary diagnosis of reaction to severe stress. Rates of depressive episodes were also low in the RHNP cohort, with the prevalence of depression in other refugee populations elsewhere being reported at 5%.^1^ The reasons discussed previously may account for this mismatch of prevalence vs service use. In addition, missing diagnoses for CMHCS clients, who may have had the above conditions, may contribute to this mismatch.

Some refugees may complement or use services of primary care physicians, family physicians, general practitioners, or specialists. However, evidence from Sydney, although somewhat dated, points to refugees using less of these services than the general Australian population.^4^ Services of general practitioners may also be sought for mental health issues, because refugees may somatize some mental health symptoms.^26^ Anecdotal evidence suggests that refugees may frequent the services of overseas-trained primary care physicians with similar sociocultural and linguistic backgrounds. The composition of refugee intake populations in high-income countries tends to change over time, and with this, the health service needs may also change.^2^ Although data linkage cannot replace surveys, it is cost-effective to use routine data linkage–based analyses to derive an overview of rates of health services contacts in the refugee population in countries with a developed data infrastructure such as Australia.^4^ Some refugees use the aforementioned STARTTS services, which may represent an additional valuable resource to study long-term use of mental health services among refugees (>3 years), whereas RHNP data are appropriate for studying use of mental health services among newly arrived refugees.

There are significant policy and research implications of this research. First, it has been established that there is a further need for understanding refugee mental health services. This understanding should encompass the entire landscape of refugee mental health services. Thus, further data linkage is required, especially to trauma services such as STARTTS and to general practitioners. A better understanding could also be obtained if refugee status were included in routine health service records, such as during visits to a general practitioner or a hospital. However, collecting this information through self-identification could lead to undercounting, because some individuals may choose to not identify as refugees after having resided for some time in Australia. The second set of implications is along the lines of intervention to better detect and treat mental health issues among refugees at the outset. Thus, it may also be advisable to screen for trauma at health service gatekeeping points using brief inventories such as the PTSD-8 inventory^27^ or the 4-item Brief Case-Find for Depression instrument.^28^ Refugees in need can then be directed to appropriate services. Finally, the need for better mental health literacy among refugees cannot be further emphasized enough. More funding should be directed toward programs to raise levels of mental health literacy among refugees and among religious leaders and priests from whom refugees seek mental health advice.^20,26^

### Limitations

Limitations of this study include the fact that nonusers of health services could not be distinguished from unsuccessful linkages. Another salient limitation is that the CMHCS data had many missing diagnoses. A possible reason is that mental health personnel in the CMHCS are generally trained to manage immediate presenting symptoms and not necessarily to diagnose conditions. Missing diagnoses render the comparison of diagnoses underpowered, which could mean that rates of the diagnosed conditions without enough numbers to reach the significance threshold could see additional cases but do not invalidate the extant significant findings. Third, because this was a data linkage study, we could not replicate the depth of information that many survey-based studies provide.^1^ Fourth, this study examines use of health services among refugees within a short period. It has been reported that in the longer term, the prevalence of trauma-related mental health problems among refugees (except for severe trauma) tends to decrease; however, use of health services increases with acculturation and education.^4^

## Conclusions

The findings of this cross-sectional study suggest that refugees who resettle in Australia tend to use fewer mental health services than the Australian general population except for specific stress-related diagnoses. However, the need for a more comprehensive analysis cannot be understated, neither can the importance of better mental health literacy and mental health screening be ignored. The present study is therefore not only an exploration of refugee mental health use but also a call for more targeted investigations.

## Notes

Supplement. **eAppendix 1.** RHNP Data **eAppendix 2.** Data Linkage and Flow, Calculating Standardized Ratios, and Diagnosis Codes
