# Supplementary material for: Exploring the Use of Hospital and Community Mental Health Services Among Newly Resettled Refugees
Source: JAMA Netw Open. 2022 Jun 2;5(6):e2212449. doi: 10.1001/jamanetworkopen.2022.12449 (PMC9163998; doi:10.1001/jamanetworkopen.2022.12449)
Supplement: Supplement. — eAppendix 1. RHNP Data eAppendix 2. Data Linkage and Flow, Calculating Standardized Ratios, and Diagnosis Codes [file jamanetwopen-e2212449-s001.pdf]

Supplementary Online Content

Mazumdar S, Chong S, Eagar S, Fletcher-Lartey S, Jalaludin B, Smith M. Exploring the use of hospital and community mental health services among newly resettled refugees. *JAMA Netw Open*. 2022;5(6):e2212449. doi:10.1001/jamanetworkopen.2022.12449

eAppendix 1. RHNP Data
eAppendix 2. Data Linkage and Flow, Calculating Standardized Ratios, and Diagnosis Codes

This supplementary material has been provided by the authors to give readers additional information about their work.

eAppendix 1. RHNP Data

To test the representativeness of these data, we compared the age, sex and COB profile of the RHNP data with refugee settlement data from the Department of Settlement Services (DSS). For this comparison, we subset the RHNP data to arrival dates in the calendar years 2013-16 and visa numbers 200 to 204 to match the DSS data. RHNP data have an overrepresentation of Iraqis and an underrepresentation of Syrians (Tables A1, A2 and A3). A figure of the study area (Figure A1) is provided below.

Table A1. Comparing DSS Data With RHNP Data, Overall Numbers, and Fractions by Country Among Females

DSS Data			RHNP Data		
IRAQ	4,550	52.43 (50.98 , 53.88)	**IRAQ**	2,569	63.70 (62.2,65.17)
SYRIAN ARAB REPUBLIC	2,821	32.51 (30.78 , 34.24)	**SYRIAN ARAB REPUBLIC**	661	16.42 (13.60 , 19.24)
AFGHANISTAN	304	3.50 (1.44 , 5.57)	**AFGHANISTAN**	261	6.48 (3.49 , 9.47)
IRAN	233	2.68 (0.61 , 4.76)	**IRAN**	134	3.33 (0.29 , 6.37)
INDIA	116	1.34 (-0.75 , 3.43)	**TIBET**	79	1.96 (-1.10 , 5.02)
LEBANON	110	1.27 (-0.82 , 3.36)	**BURMA**	74	1.84 (-1.22 , 4.90)
OTHER	544	6.27 (4.23 , 8.31)	**OTHERS**	255	6.32 (5.61, 7.11)

Abbreviations: DSS: Department of Social Services, RHNP: Refugee Health Nurse Program

Table A2. Comparing DSS Data With RHNP Data, Overall Numbers, and Fractions by Country Among Males

DSS Data			RHNP Data		
IRAQ	4,249	49.77 (48.26 , 51.27)	**IRAQ**	2,490	61.86 (60.35, 63.35)
SYRIAN ARAB REPUBLIC	2,832	33.17 (31.44 , 34.90)	**SYRIAN ARAB REPUBLIC**	594	15.06 (12.18 , 17.94)
AFGHANISTAN	411	4.81 (2.74 , 6.88)	**AFGHANISTAN**	255	6.47 (3.45 , 9.49)
IRAN	249	2.92 (0.83 , 5.01)	**IRAN**	155	3.93 (0.87 , 6.99)
INDIA	120	1.41 (-0.70 , 3.51)	**TIBET**	129	3.27 (0.20 , 6.34)
TIBET (SO STATED)	106	1.24 (-0.87 , 3.35)	**BURMA**	80	2.03 (-1.06 , 5.12)
OTHER	571	6.69 (4.64 , 8.74)	**OTHERS**	241	2.95 (2.60, 3.34)

© 2022 Mazumdar S et al. *JAMA Network Open*.

Abbreviations: DSS: Department of Social Services, RHNP: Refugee Health Nurse Program

The RHNP data were subset to the following visa categories- 200, 201, 202, 203 and 204. The five visa classes are

- Refugee (subclass 200): This visa is for those individuals who have been referred by the United nations High Commissioner for Refugees to Australia for resettlement
- In-Country Special Humanitarian (subclass 201): This people is for individuals still living in their country and are unable to leave
- Global Special Humanitarian visa (subclass 202): This visa is meant for individuals who face persecution in their home country but are not registered as refugees with the United nations High Commissioner for Refugees
- Emergency Rescue (subclass 203): This visa is for those individuals who have been referred by the United nations High Commissioner for Refugees to Australia for resettlement and face immediate danger
- Woman at Risk (subclass 204): This visa is for women who do not have the protection of a partner or relative and are danger of victimisation

Further these data were subset to arrival dates between 1 Jan 2013 and 21 Dec 2016 to match the available DSS data. The RHNP data have a slight overrepresentation of Iraqis. Beyond the top four sending countries (Iraq, Syrian Arab Republic, Afghanistan and Iran, the numbers are too small for valid comparisons). Note that 35 people did not have any gender identified.

© 2022 Mazumdar S et al. *JAMA Network Open*.

Table A3. Comparing DSS Data With RHNP Data, Comparing Sex Compositions and Country of Birth#

	Percent Female in the overall refugee population from a given country			
	**DSS**			**RHNP**
IRAQ	51.71			50.53
SYRIAN ARAB REPUBLIC	49.90			52.67
AFGHANISTAN	42.52			50.19
IRAN	48.34			46.37
	**Percent Male in the overall refugee population from a given country**			
IRAQ	48.29			48.98
SYRIAN ARAB REPUBLIC	50.10			47.33
AFGHANISTAN	57.48			49.04
IRAN	51.66			53.63

Abbreviations: DSS: Department of Social Services, RHNP: Refugee Health Nurse Program

Note that country of birth here is as recorded in DSS and RHNP datasets. Given the complex nature of the refugee journey, this may not always represent the actual sending country from which the refugee arrives in Australia.

© 2022 Mazumdar S et al. *JAMA Network Open*.

Table A4. Comparing the Australian Population With the RHNP Population by Age and Sex

The tables below provide comparisons between the RHNP cohort (RHNPc) and the Australian population in 2016. The Australian population is older in comparison to the RHNPc population.

	RHNPc	Australian Population
**Age Group**	**Percent**	**Percent**
0 to 9	17	12.7
10 to 19	20	12
20 to 29	18	13.8
30 to 39	15	14
40 to 49	13	13.5
50 to 59	9	12.7
60 to 69	5	10.6
70 to 79	3	6.6
80 and older	1	4

	RHNPc	Australian Population
**Gender**	**Percent**	**Percent**
Male	49	49
Female	51	51

© 2022 Mazumdar S et al. *JAMA Network Open*.

Figure A1. Locations of Refugee Health Nurse Program Clinics in Sydney Metropolitan Area Relative to the Clientele Population (2012-2016)

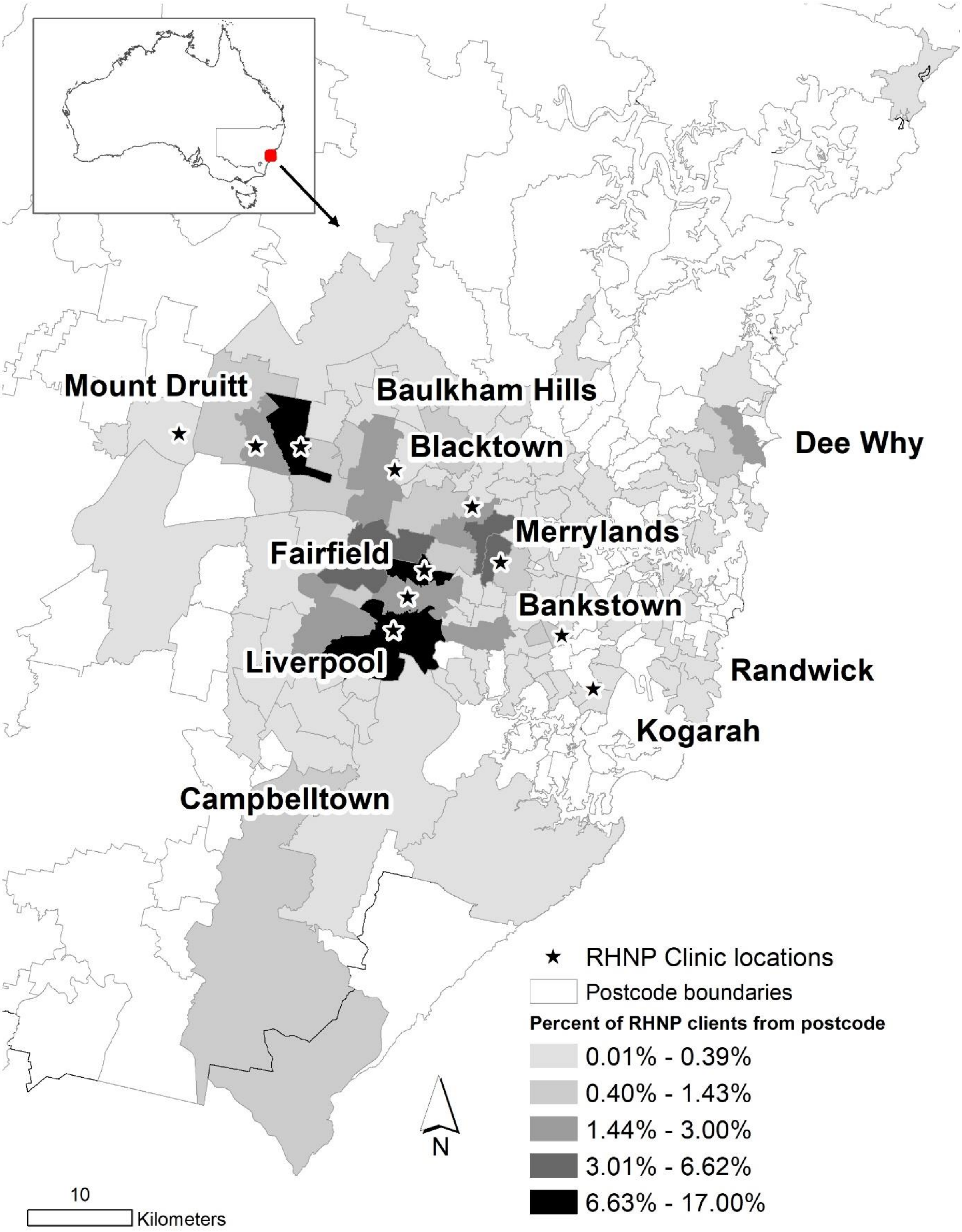

© 2022 Mazumdar S et al. *JAMA Network Open*.

eAppendix 2. Data Linkage and Flow, Calculating Standardized Ratios, and Diagnosis Codes

Data Linkage and Data Flow

The linkage was performed by the Centre for Health Record Linkage (CHeReL), NSW Ministry of Health, using probabilistic record linkage, personal identifiers and de-identification[6]. Data linkage resulted in a total of 7,023 APDC records representing 2,778 people with both mental health and other hospitalisations, and 7,538 MH-AMB records representing 303 people. CHeReL's methods of data linkage are validated, are widely used for research, and have a low linkage error rate of 0.05 %[7]. We implemented a validation to check concordance between the age, sex and COB in the RHNP data records with the MH-AMB records in the linked data. Refugee age can be inaccurate for a number of reasons, including poor recording in source countries and loss of documentation. As such, the median difference in age recorded in the two datasets was 108 days, the sex concordance was 96%, and 87% of the recorded COB were the same. Among the 35 discordant COB records, 16 were because of refugees changing their country of birth to Australia.

For all analyses or statistics using linked data, we used the date that the first client visited the RHNP clinics (in our dataset) and the date the last client visited as censoring dates. Thus the first service contact in the CMHCS data and the date of admission in the APDC data were censored to 'on or after 23rd October 2012'. The corresponding endpoint date was 8th June 2017. This reduced the number of people in the linked CMHCS data to 265. A further nine people in the CMHCS data had missing diagnosis and one outlier person with an excessive number of service contacts (n=463) was removed, resulting in 255 people in the linked CMHCS-RHNP data. Thus, our CMHCS analyses were based on these 255 people. A similar data flow process on the APDC data resulted in a final linked APDC dataset representing 115 mental health hospitalisations representing 71 people. A data flowchart is provided below, Figure A2.

© 2022 Mazumdar S et al. *JAMA Network Open*.

Figure A2. Data Flowchart

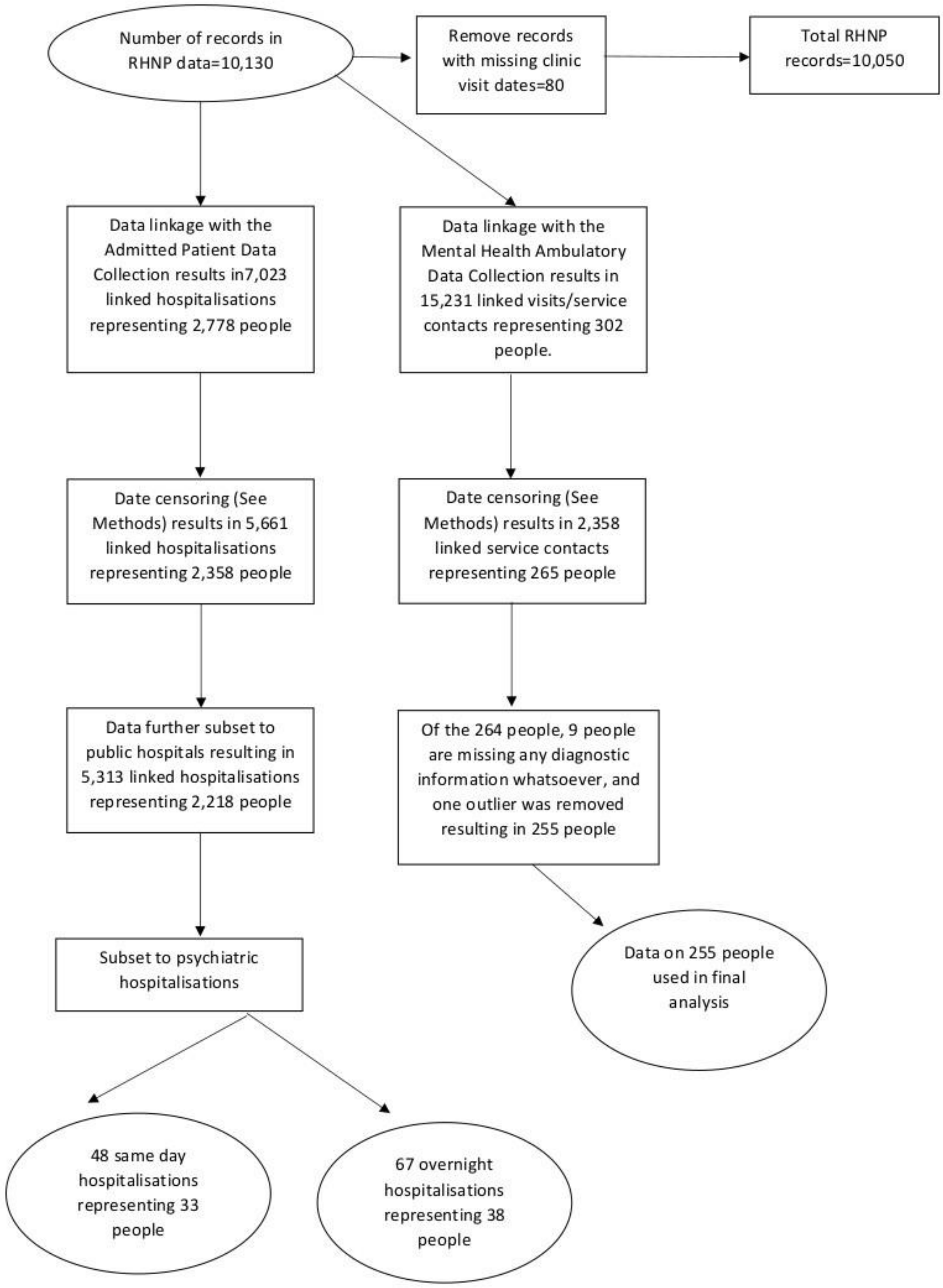

© 2022 Mazumdar S et al. *JAMA Network Open*.

Calculating Standardized Ratios

Expected number of service contacts were derived by calculating the crude rates for a given category in the Australian population and multiplying the total person years in the specific age or sex category in the RHNPc by this rate. Total person years in the specific age or sex category were in turn calculated by deriving the number of days each person in RHNPc existed in the study window (arrival to end point of study), dividing by 365, and then summing across specific age or sex categories. Thus, for example the 18-34 age group in RHNPc contributes 2,902 person years, calculated by summing person year contributions by each person in that group. The entire RHNPc comprised a total of 25,028 person years, and this formed the basis for ICD10 diagnosis comparisons. If a particular ICD10 diagnoses resulted in less than 100 service contacts, they were removed. Crude rates in the Australian population were calculated using four years of data for age and sex comparisons and one year of data for ICD 10 diagnosis comparisons, the difference being caused by AIHW data availability constraints[6].

© 2022 Mazumdar S et al. *JAMA Network Open*.

***ICD-10-AM* diagnosis codes used to define mental health-related hospital separations**

F00	F28	F62	F95	Z00.4
F01	F29	F63	F98(c)	Z03.2
F02	F30	F64	F99	Z04.6
F03	F31	F65	G30.0	Z09.3
F04	F32	F66	G30.1	Z13.3
F05	F33	F68	G30.8	Z50.2
F06	F34	F69	G30.9	Z50.3
F07	F38	F70	G47.0	Z54.3
F09	F39	F71	G47.1	Z61.9
F10	F40	F72	G47.2	Z63.1
F11	F41	F73	G47.8	Z63.8
F12	F42	F78	G47.9	Z63.9
F13	F43	F79	O99.3	Z65.8
F14	F44	F80	R44.0	Z65.9
F15	F45	F81	R44.1	Z71.4
F16	F48	F82	R44.2	Z71.5
F17	F50	F83	R44.3	Z76.0
F18	F51	F84(b)	R44.8	
F19	F52(a)	F88	R45.0	
F20	F53	F89	R45.1	
F21	F54	F90	R45.4	
F22	F55	F91	R48.0	
F23	F59	F92	R48.1	
F24	F60	F93	R48.2	
F25	F61	F94	R48.8	

Obtained from:

https://www.aihw.gov.au/reports/mental-health-services/mental-health-services-in-australia/classifications-and-technical-notes

© 2022 Mazumdar S et al. *JAMA Network Open*.
